# Chronic otitis media is initiated by a bulla cavitation defect in the FBXO11 mouse model

**DOI:** 10.1242/dmm.038315

**Published:** 2019-03-21

**Authors:** Jorge del-Pozo, Neil MacIntyre, Ali Azar, James Glover, Elspeth Milne, Michael Cheeseman

**Affiliations:** 1 Veterinary Pathology, The Royal (Dick) School of Veterinary Studies, University of Edinburgh, Edinburgh EH25 9RG, UK; 2 Developmental Biology Division, Roslin Institute and The Royal (Dick) School of Veterinary Studies, University of Edinburgh, Edinburgh EH25 9RG, UK; 3 Centre for Comparative Pathology & Division of Pathology, University of Edinburgh, Institute of Genetics & Molecular Medicine, Crewe Road, Edinburgh EH4 2XR, UK

**Keywords:** BPIFA1, Neural-crest-derived epithelium, Keratins 5, 8, 7 and 19, BCL6, SNAI1

## Abstract

Auditory bulla cavitation defects are a cause of otitis media, but the normal cellular pattern of bulla mesenchyme regression and its failure are not well understood. In mice, neural-crest-derived mesenchyme occupies the bulla from embryonic day 17.5 (E17.5) to postnatal day 11 (P11) and then regresses to form the adult air-filled bulla cavity. We report that bulla mesenchyme is bordered by a single layer of non-ciliated epithelium characterized by interdigitating cells with desmosome cell junctions and a basal lamina, and by *Bpifa1* gene expression and laminin staining of the basal lamina. At P11-P12, the mesenchyme shrinks: mesenchyme-associated epithelium shortens, and mesenchymal cells and extracellular matrix collagen fibrils condense, culminating in the formation of cochlea promontory mucosa bordered by compact non-ciliated epithelial cells. *FBXO11* is a candidate disease gene in human chronic otitis media with effusion and we report that a bulla cavitation defect initiates the pathogenesis of otitis media in the established mouse model *Jeff* (*Fbxo11^Jf/+^*). Persistent mesenchyme in *Fbxo11^Jf/+^* bullae has limited mesenchymal cell condensation, fibrosis and hyperplasia of the mesenchyme-associated epithelium. Subsequent modification forms fibrous adhesions that link the mucosa and the tympanic membrane, and this is accompanied by dystrophic mineralization and accumulation of serous effusion in the bulla cavity. Mouse models of bulla cavitation defects are important because their study in humans is limited to post-mortem samples. This work indicates new diagnostic criteria for this otitis media aetiology in humans, and the prospects of studying the molecular mechanisms of murine bulla cavitation in organ culture.

## Abstract (precis)

**Summary:** FBXO11 is a candidate disease gene for otitis media in humans and the authors report that a bulla cavitation defect initiates otitis media in the *Fbxo11^Jf/+^* mouse model.

## INTRODUCTION

Auditory bulla cavitation, or pneumatization, in the mouse involves the regression of embryonic mesenchyme by postnatal day (P) 11 to form the adult air-filled bulla (Park and Lim, 1992a), allowing the ossicles to move freely and transmit sound from the tympanic membrane to the cochlea. Lineage-tracing experiments show that the epithelium that lines the middle ear mucosa has a dual origin. A ciliated pseudostratified epithelium with goblet cells lines the auditory tube and adjacent bulla surface and has an endodermal origin, whereas the non-ciliated epithelium lining the attic and cochlea promontory is neural-crest derived, and is formed by a mesenchymal–epithelial transition (MET) a few days after bulla cavitation (Thompson and Tucker, 2013). The dorsal pole of the bulla adjacent to the round window is also lined with a ciliated epithelium and the polarity of ciliated cells throughout the bulla is aligned such that beating is coordinated towards the auditory-tube opening (Luo et al., 2017). Disruption of the bulla cavitation can impair middle ear function. For instance, individuals with Treacher-Collins syndrome with mutations in the *TCOF* gene, encoding the nucleolar phosphoprotein treacle, have hearing loss and, in the *Tcof1^+/−^* mutant mouse, retention of bulla mesenchyme is associated with conductive hearing loss and limits the growth of the adult bulla (Richter et al., 2010). Retention of bulla mesenchyme, particularly when it is associated with the mastoid air cells, is also a risk factor for inflammation of the middle ear (otitis media) in humans (Jaisinghani et al., 1999) and, in the *Cdh11* knockout (KO) mouse, retention of mesenchyme is associated with hearing deficits, reduced bulla size and otitis media (Kiyama et al., 2018).

*FBXO11* is a candidate disease gene in human chronic otitis media with effusion (Segade et al., 2006; Rye et al., 2011a,b; Bhutta et al., 2017a,b; also see reviews by Rye et al., 2011a,b; Rye et al., 2012) and the *Jeff* (*Fbxo11^Jf/+^*) mutant mouse (Mouse Genome Informatics number 1862017) has conductive hearing loss and chronic otitis media (Hardisty et al., 2003; Hardisty-Hughes et al., 2006). FBXO11 is a member of the FBXO family of proteins that bind target proteins for ubiquitination and proteasomal degradation (Skaar et al., 2013; Cardozo and Pagano, 2004). The Q491L *Fbxo11^Jf/+^* mutation (Hardisty-Hughes et al., 2006) is located in the presumptive substrate-binding domain of the FBXO11 protein (Skaar et al., 2013). Its cargoes include the transcription factors BCL6 (Schneider et al., 2016) and SNAI1/SNAI2 (Díaz and de Herreros, 2016; Jin et al., 2015). FBXO11 neddylates and inactivates p53 (Abida et al., 2007), promotes degradation of cell-cycle regulator Cdt2 to stimulate *in vitro* epithelial cell migration (Abbas et al., 2013) and has arginine methyltransferase enzyme activity, which regulates hypoxia-inducible factor HIF-1α (Ju et al., 2015). Homozygous mutation in *Fbxo11^Jf/Jf^* mice modulates TGFβ signalling via interaction with p53 and increases nuclear localization of phospho-Smad2 (pSmad2) to disrupt embryonic epithelial closure mechanisms in the palate and eyelid, and in airway branching in the lung (Tateossian et al., 2009, 2015). Recent work shows that *FBXO11* mutations (Online Mendelian Inheritance in Man number 607871) are associated with intellectual disability (Fritzen et al., 2018) and variable neurodevelopmental disorder (Gregor et al., 2018); it is noteworthy that 3 of 20 children in the latter cohort had otitis media, but a background level of spontaneous chronic otitis media might be expected in this age group.

FBXO11 protein is expressed in middle ear pseudostratified epithelium of wild-type mice from embryonic day (E) 18.5 to P21 (Hardisty-Hughes et al., 2006), but the role of the *Fbxo11^Jf/+^* mutation in otitis media pathogenesis remains unclear. Auditory-tube abnormalities were reported in newborn and adult *Fbxo11^Jf/+^* mice as well as the occurrence of scattered calcification of the bulla mucosa at 11 months of age (Hardisty et al., 2003). The other histopathological changes reported in this work, such as polyp formation, hyperplasia of ciliated epithelial cells and exudation into the bulla cavity, are common in chronic otitis media in mouse genetic models (Bhutta et al., 2017a,b). Thickening of the underlying bulla bone (osteosclerosis) (Hardisty et al., 2003) is a common response to injury and together these secondary changes do not suggest a specific initiating cause of otitis media.

In this work, we have investigated the normal process of bulla cavitation and report that defective bulla cavitation is the initiating event in otitis media pathogenesis in *Fbxo11^Jf/+^* mice.

## RESULTS

### Embryonic and neonatal bulla mesenchyme has an epithelial margin

**Fig. 1.**
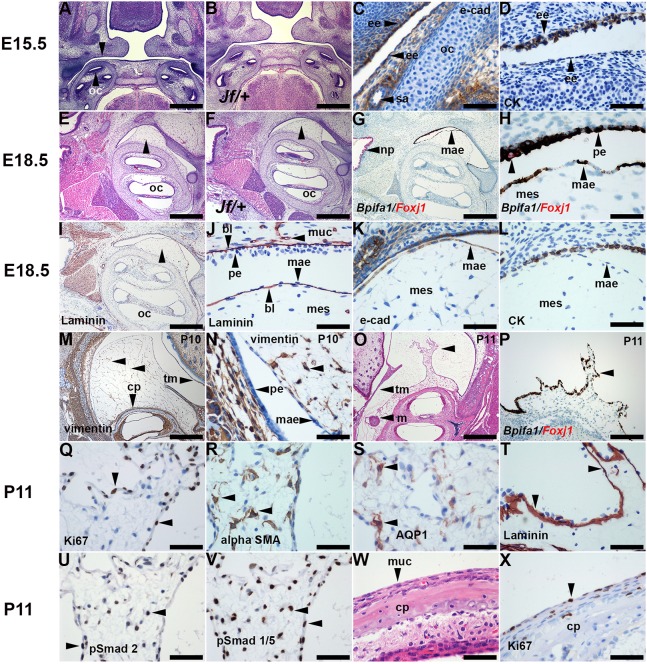
**Regression of bulla mesenchyme in wild-type mice.** (A,C,D) E15.5 *Fbxo11^+/+^* (mixed C57BL/6J C3H background) and (B) *Fbxo11^Jf/+^* mice have a first pharyngeal pouch lined with endoderm-derived epithelium (unlabelled arrowhead in A), E-cadherin and (D) wide-spectrum cytokeratin IHC. (E,G-L) E18.5 *Fbxo11^+/+^* and (F) *Fbxo11^Jf/+^*. (E,F) Bulla mesenchyme (arrowheads) projects from the otic capsule. (G,H) Duplex ISH *Bpifa1* signals are brown spots and *Foxj1* signals are red spots. (G) Nasopharynx ciliated epithelium has a *Foxj1* signal. (H) Higher magnification of mesenchyme-associated epithelium (MAE) and pseudostratified mucosal epithelium shows an intense *Bpifa1* signal. Occasional *Foxj1*-positive ciliated cells are detected in mucosal epithelium (single unlabelled arrowhead indicates a *Foxj1*-positive cell). (I) Epithelia, muscle and blood vessels have a basal lamina that stains for laminin. (J) Higher magnification of mesenchyme, MAE and pseudostratified mucosal epithelium. Note laminin staining of capillary (horizontal arrowhead) as well as epithelial basal lamina. (K) The MAE stains for E-cadherin, (L) but wide-spectrum cytokeratin staining is absent. (M,N) P10 C57BL/6J vimentin IHC. (M) Bulla mesenchyme has vimentin-positive mesenchymal cells with cytoplasmic projections that form an interconnecting meshwork (unlabelled arrowheads). (N) Higher magnification of panel M shows that the MAE and mucosal epithelium are vimentin negative. (O-X) P11 C57BL/6J. (O,P) Two examples of partially regressed mesenchyme with irregular spiky projections (unlabelled arrowheads); (O) H&E, (P) *Bpifa1/Foxj1* ISH. (Q-V) Partly regressed mesenchyme; (Q) Ki67 IHC showing a high proliferation index in MAE. (R) αSMA and (S) AQP1 are expressed in mesenchymal cells; (T) the laminin-positive basal lamina remains in place. (U) pSmad2 and (V) pSmad1/5 are widely expressed during regression. (W,X) P11 C57BL/6J promontory mucosa with non-ciliated epithelium, which in X has a moderate Ki67 index. bl, basal lamina; CK, wide-spectrum cytokeratin IHC; cp, cochlea promontory; ee, endoderm-derived epithelium; e-cad, E-cadherin IHC; m, malleus; mae, mesenchyme-associated epithelium; mes, mesenchyme; muc, mucosa; oc, otic capsule; np, nasopharynx; pe, pseudostratified epithelium; sa, stapedial artery; tm, tympanic membrane. Scale bars: (A,B,E-G,I,M,O) 500 µm; (P) 200 µm; (C,D,H,J-L,N,Q-X) 50 µm.

The cellular organization of normal bulla mesenchyme is not well documented, so we performed a time-course study in wild-type mice as well as in *Fbxo11^Jf/+^* mice. E15.5 heads have bilateral outgrowths of pharyngeal endoderm (first pharyngeal pouches) (Fig. 1). The bulla mesenchyme arises over the otic capsule and its histology appears similar in E17.5-P10 *Fbxo11^Jf/+^* and *Fbxo11^+/+^* bullae (Fig. 1). Embryonic and postnatal mesenchyme is bordered by a single layer of squamous cells that shows immunohistochemistry (IHC) staining for E-cadherin, but IHC staining with an anti-wide-spectrum-cytokeratin antibody was weak or absent (Fig. 1). A number of keratins are produced by primary cultures of mouse middle ear epithelial cells (Mulay et al., 2016). Among these, K8 is a primary keratin and K7/K19 are secondary keratins of simple epithelial cells (Moll et al., 2008). K5 is principally expressed in basal cells of endodermal-derived pseudostratified epithelium (Tucker et al., 2018). We found that squamous cells showed IHC staining with K7/K19 and K8, as well as K5 (Fig. S1).

Bpifa1 (also known as Splunc1) is one of the most abundant secretory proteins in the upper respiratory tract (Musa et al., 2012; Mulay et al., 2016, 2018) and the mesenchyme border has an intense *Bpifa1 in situ* hybridization (ISH) signal (Fig. 1). Hereafter, we designate this layer as the mesenchyme-associated epithelium (MAE). *Bpifa1* ISH signals are also present in the bulla mucosa epithelium (but not in the medial tympanic membrane epithelium), whereas the ciliated cell marker *Foxj1* is strongly expressed in the nasopharynx and auditory-tube epithelium and focally expressed in bulla mucosal epithelium (Fig. 1, Fig. S2). The MAE has a laminin-positive basal lamina (Fig. 1). The centre of the mesenchymal mass has loosely packed spindle and stellate cells that stain for the mesenchymal marker vimentin, but the MAE is vimentin negative (Fig. 1).

**Fig. 2.**
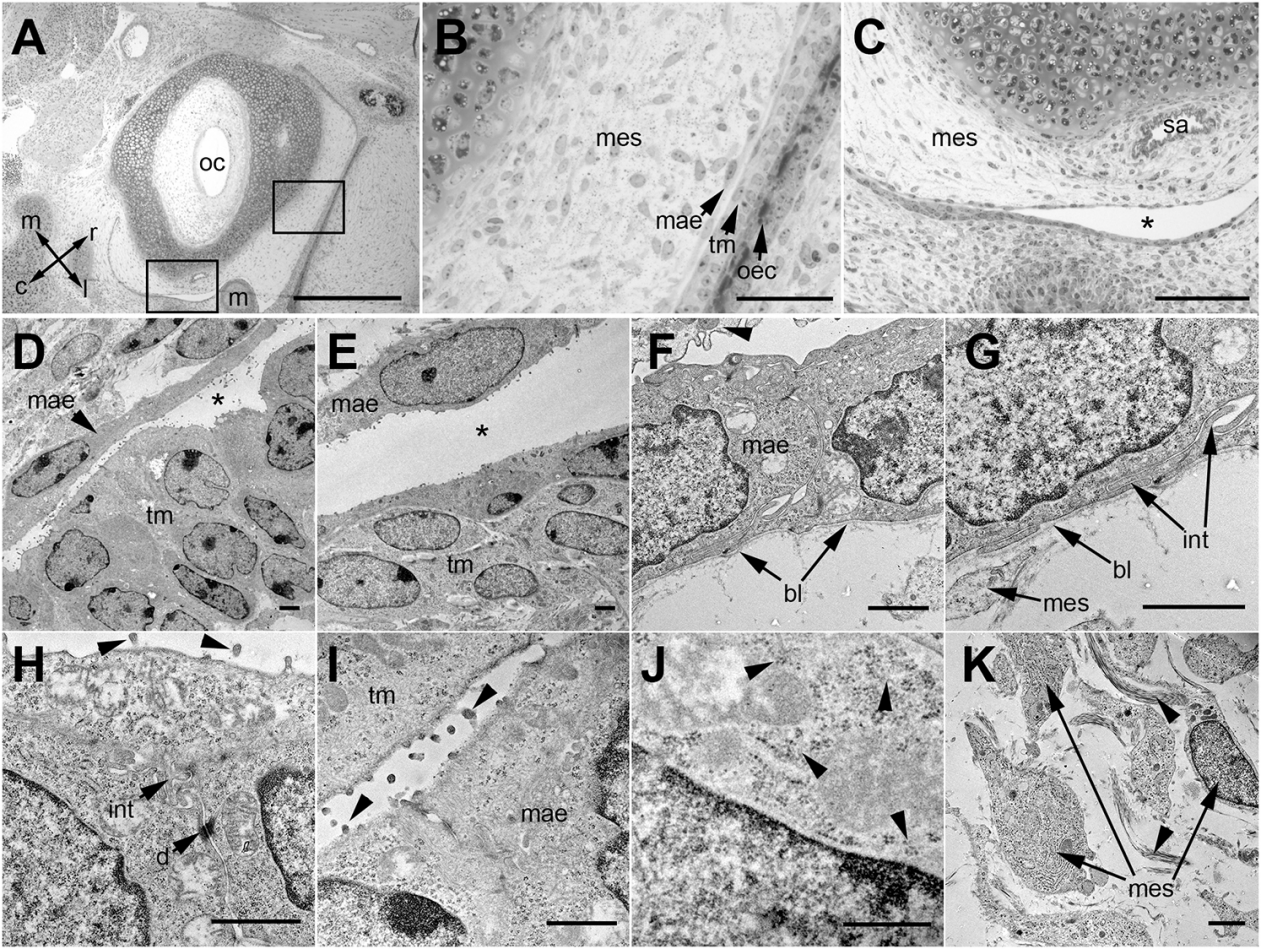
**The ultrastructure of P0 C57BL/6J bulla mesenchyme shows a squamous epithelial margin.** (A-C) Semi-thin Toluidine-Blue-stained sections showing bulla and otic capsule with bulla mesenchyme bordered by a single layer of epithelium. (A) Rectangles indicate location of panels B and C; arrow directions indicate the orientation: rostral (r), caudal (c), medial (m), lateral (l). (B) The mesenchyme is bordered by a single-layered epithelium that closely abuts the multi-layered epithelium of the tympanic membrane epithelium and outer canal. (C) A cleft (asterisk) separates the mesenchyme and facing multi-layered epithelium. (D-K) Transmission electron microscopy. (D,E) The mesenchyme-associated epithelium (MAE) and the facing multi-layered epithelium is separated by a variably wide cleft (asterisk). (F) MAE rests on a basal lamina and has an overlying effete cell (arrowhead) on its apical surface. (G) Higher magnification of panel F shows that the basal region of the MAE cell has a basal lamina and interdigitating cytoplasmic projections with a neighbouring epithelial cell. (H,I) Apical regions of the MAE cell show interdigitations, desmosome cell junctions and filopodia (arrowheads) projecting from the apical cell surface. (J) The MAE has cytoplasmic glycogen granules (arrowheads). (K) Mesenchymal cells and scattered bundles of collagen fibrils (arrowheads) in the ECM. bl, basal lamina; d, desmosome cell junction; int, interdigitation of adjoining epithelial cells; m, malleus; mae, mesenchyme-associated epithelium; mes, mesenchyme; oec, outer ear canal epithelium; oc, otic capsule; sa, stapedial artery; tm, tympanic membrane epithelium. Scale bars: (A) 500 µm, (B) 50 µm, (C) 100 µm, (D-K) 1 µm.

The ultrastructural features of bulla mesenchyme in P0 C57BL/6J mice are similar to those described in the rat (Hilding et al., 1980). The border of the mesenchyme has a single layer of interdigitating epithelial cells with desmosome junctions and a basal lamina. Apical surfaces of the MAE and the opposing multi-layered epithelium of the tympanic membrane have filopodia. The mesenchymal cells are widely spaced and the extracellular matrix (ECM) has collagen fibrils (Fig. 2).

### Bulla mesenchyme regression in wild-type mice

**Fig. 3.**
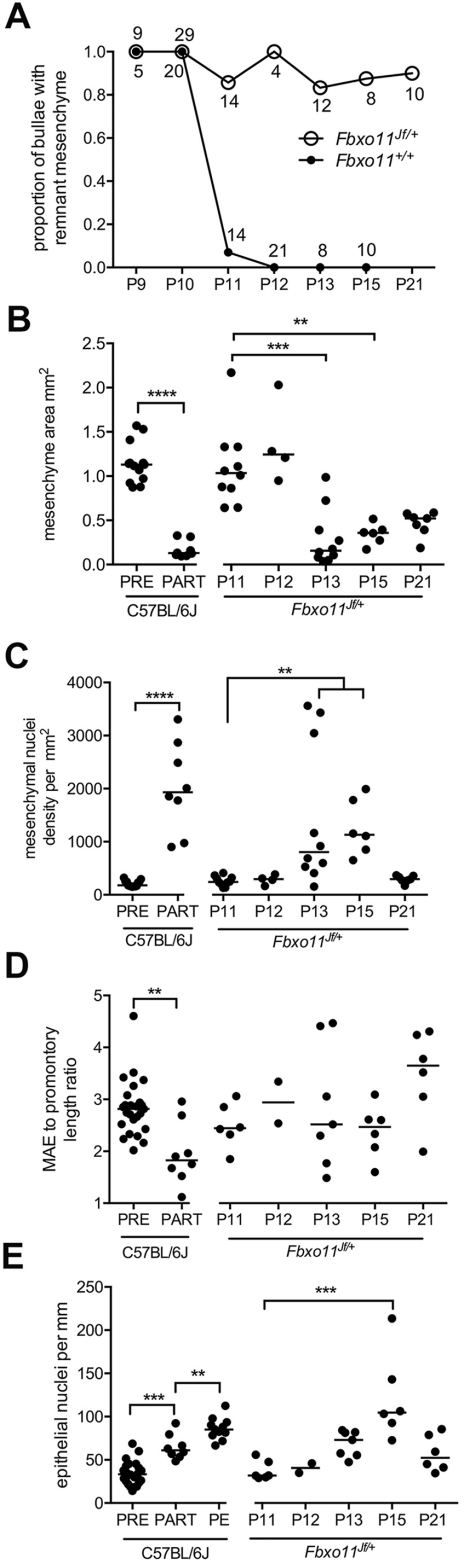
**Bulla mesenchyme condensation.** (A) Bulla mesenchyme regresses after P10 in *Fbxo11^+/+^* but persists in *Fbxo11^Jf/+^* mice. Numbers adjacent to points indicate the number of bullae examined; numbers above the symbol are for *Fbxo11^+/+^* and those beneath are for *Fbxo11^Jf/+^* bullae. (B) Mesenchyme area and (C) mesenchymal nuclei density. C57BL/6J partially regressed mesenchyme (PART) has a reduced area compared with pre-regression mesenchyme (PRE), and its mesenchymal nuclei density is increased. Remnant P13 and P15 *Fbxo11^Jf/+^* mesenchyme shrinks and has increased nuclei density. (D) MAE to cochlea promontory length ratios and (E) epithelial nuclei densities. C57BL/6J partially regressed mesenchyme epithelium is shorter than pre-regression mesenchyme epithelium. C57BL/6J epithelial density increases from pre- to partially regressed mesenchyme and to promontory epithelium (PE). *Fbxo11^Jf/+^* bullae have persistent MAE and epithelial density is significantly increased at P15. Data in histograms (B-E) are represented as points and the median. Statistical tests were chosen after performing D'Agostino and Pearson omnibus normality tests. C57BL/6J data were analyzed with Student's *t*-tests, or ANOVA with Tukey's multiple comparison tests (except that MAE ratios were analyzed with a Mann–Whitney test). *Fbxo11^Jf/+^* data were analyzed with Kruskal–Wallis tests and Dunn's multiple comparisons using P11 data as the reference control. Two-tailed tests: **P<0.01, ***P<0.001, ****P<0.0001; all other comparisons were not statistically significant.

**Fig. 4.**
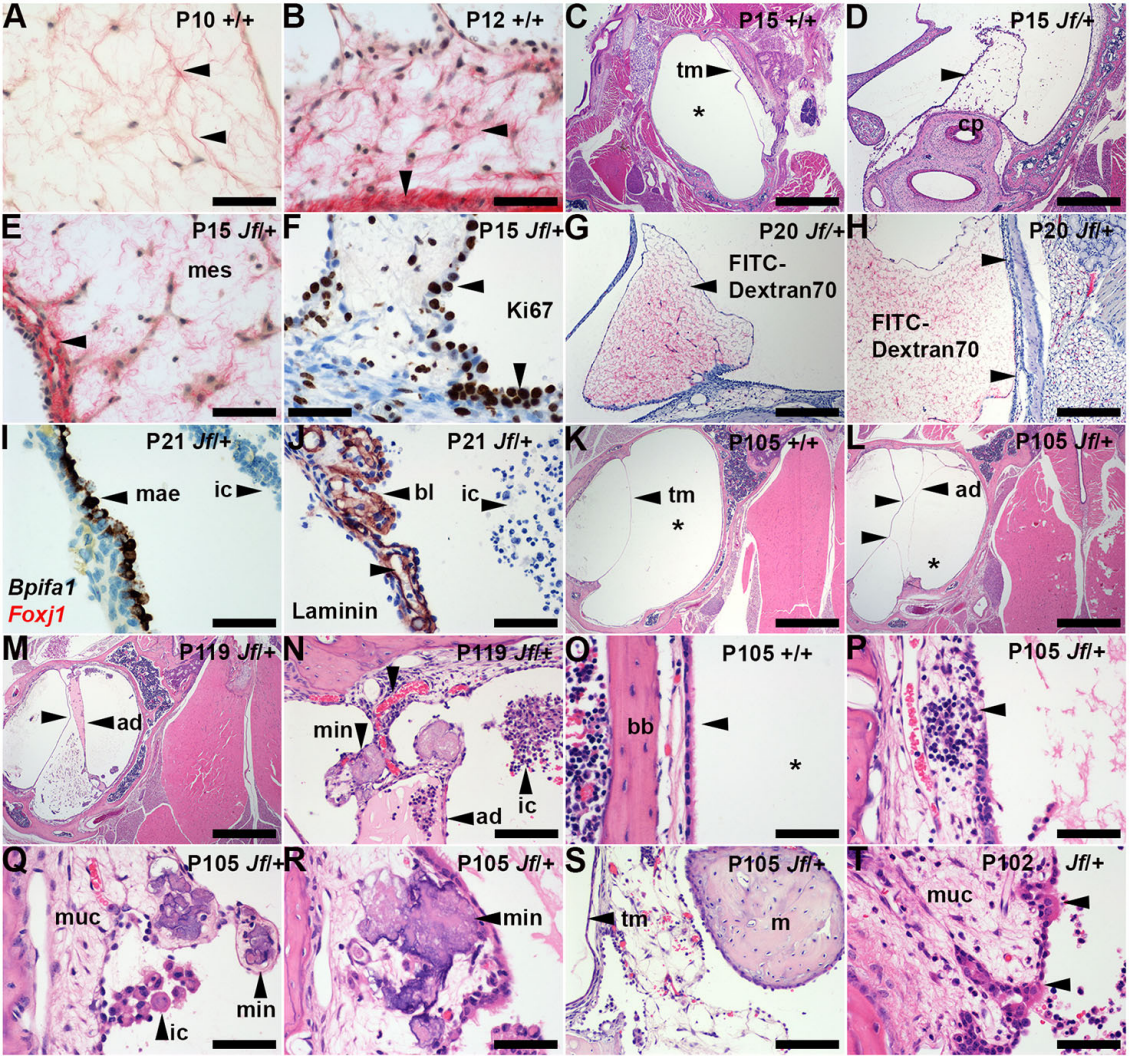
**Otitis media in adult *Fbxo11^Jf/+^* mice is characterized by bulla adhesions and soft-tissue mineralization.** (A) P10 C57BL/6J pre-regression mesenchyme has widely spaced mesenchymal cells and Picrosirius-Red-stained collagen fibrils are in loose arrays (arrowheads). (B) P12 C57BL/6J partially regressed mesenchyme in which ECM collagen fibrils are more closely clustered (horizontal arrowhead); the periosteal connective tissue has a dense band of collagen (vertical arrowhead). (C) P15 *Fbxo11^+/+^* bulla with normal air-filled bulla cavity (asterisk). (D-F) P15 *Fbxo11^Jf/+^* remnant MAE projecting from the cochlea promontory (arrowhead in D); (E) collagen fibrils are clustered around mesenchymal cells and beneath the MAE (arrowhead) and (F) Ki67 staining shows a high proliferation rate and the epithelial cell density is focally hyperplastic (arrowheads). (G,H) P20 *Fbxo11^Jf/+^* remnant mesenchyme labelled *in vivo* with FITC­–dextran-70 shows red staining of mesenchyme ECM (arrowheads); (H) remnant mesenchyme lies against the bulla mucosa. (I,J) P21 *Fbxo11^Jf/+^* MAE shows *Bpifa1* ISH signals (but not *Foxj1* signals); (J) laminin-stained basal lamina of MAE and capillaries (unlabelled arrowhead). (K-T) P105-P119 *Fbxo11* bullae. (K) Normal healthy *Fbxo11^+/+^* bulla with a curved tympanic membrane profile and air-filled bulla cavity (asterisk). (L-N) *Fbxo11^Jf/+^* bullae with adhesions that attach mucosa to tympanic membrane and indent its profile (unlabelled arrowheads in L and M); (L) inflammatory cell effusion in the bulla cavity is minimal, whereas (M) cross-linking of adhesions create compartments containing effusions of different cellularity and eosin staining intensity. (N) A higher magnification of panel M shows an adhesion attachment point with a vascular pedicel and a capillary blood vessel (unlabelled arrowhead) and mineralized foci. (O) Normal *Fbxo11^+/+^* bulla mucosa with a ciliated epithelium (arrowhead) and air-filled bulla cavity (asterisk). (P) *Fbxo11^Jf/+^* thickened mucosa with lymphoid infiltrates (arrowhead). (Q,R) Mucosal mineralization in (Q) non-ciliated and (R) ciliated epithelial regions (arrowheads). (S) Mesenchyme remnant attached to tympanic membrane; (T) *Fbxo11^Jf/+^* promontory epithelium has small populations of ciliated cells (arrowheads). ad, adhesion; bl, basal lamina; bb, bulla bone; cp, cochlea promontory; ic, inflammatory cells; m, malleus; mae, mesenchyme-associated epithelium; mes, mesenchyme; min, mineralization; muc, mucosa; tm, tympanic membrane. Scale bars: (C,K-M) 1000 µm; (D) 500 µm; (G,H) 200 µm; (N,S) 100 µm; (A,B,E,F,I,J,O-R,T) 50 µm.

We next investigated the process of normal mesenchyme regression in wild-type mice in the critical period from P10 onwards. The P10 cochlea promontory has a projecting mass of mesenchyme that is replaced by a slender mucosa with a non-ciliated epithelium by P12. One possible instance of transition from mesenchyme to promontory mucosa was observed in 76 P10-P12 *Fbxo11^+/+^* bullae, where the mucosa was slightly thickened with rarefied connective tissue and light hemorrhage. We collected 69 P10-P12 C57BL/6J mice and found that 8/94 bullae in the P11-P12 age group had more convincing examples of mesenchyme regression characterized by shrunken and spiky profiles (Fig. 1). Partially regressed mesenchyme had increased mesenchymal cell density (Fig. 3), a steady cell population size (a median of ∼210 in pre- and partially regressed mesenchyme) and increased ECM collagen fibril density (Fig. 4). The MAE shortens as it transitions from pre-regression to partially regressed mesenchyme and on to promontory epithelium. As this occurs, the MAE, but not the mesenchyme (Fig. 1Q), has a high Ki67 proliferation index, the cell density increases (Fig. 3) and the basal lamina remains in place (Fig. 1). *Bpifa1* was expressed in partially regressed MAE and in promontory epithelium, but these epithelia did not have *Foxj1*-positive ciliated cells (Fig. S2). The newly formed promontory epithelium in P13 *Fbxo11^+/+^* bullae showed uniform K7/K19 and K8 expression, whereas K5 was expressed in a subpopulation of cells (Fig. S3).

Mesenchymal cell AQP1 and αSMA staining was strong in partially regressed tissue, whereas nuclear localization of pSmad2 and pSmad1/5 was widespread in pre-regression and regressing mesenchyme (Fig. 1), and in bulla mucosa. Scattered macrophages were present pre-regression and in regressing mesenchyme, but there was no evidence of apoptosis.

### Mesenchyme regression is delayed in *Fbxo11^Jf/+^* mice

Establishing the normal pattern and timing of mesenchyme regression in wild-type mice enabled us to identify key differences in *Fbxo11^Jf/+^* bullae. In contrast to wild-type mice, >80% of P11-P21 *Fbxo11^Jf/+^* bullae had persistent mesenchyme (Fig. 3). Retained *Fbxo11^Jf/+^* MAE fails to shorten and, in a minority of cases, there is increased epithelial cell density and localized hyperplasia (Figs 3,4). As well as *Bpifa1* gene expression (Fig. 3), the P13 remnant MAE shows IHC staining with K7/K19, K8 and K5 (Fig. S3).

AQP1 and αSMA were expressed in remnant *Fbxo11^Jf/+^* mesenchyme cells. Collagen fibrils clustered in a sub-epithelial band and around mesenchymal cells (Fig. 4). Nuclear localization of pSmad2 and pSmad1/5 was comparable in *Fbxo11^+/+^* and *Fbxo11^Jf/+^* at P10, and was also widespread in retained P13-P15 *Fbxo11^Jf/+^* mesenchymal cells and MAE. Macrophage numbers were low and there were no detectable apoptotic cells. The vascular permeability marker fluorescein isothiocyanate (FITC)–dextran-70 labels the ECM of mesenchyme remnant tissue in P20 *Fbxo11^Jf/+^* mice, and contact between mesenchyme and mucosa (Fig. 4) and tympanic membrane may be the origin of adhesions (see below).

### Otitis media in *Fbxo11^Jf/+^* mice is characterized by adhesions and soft-tissue mineralization

**Fig. 5.**
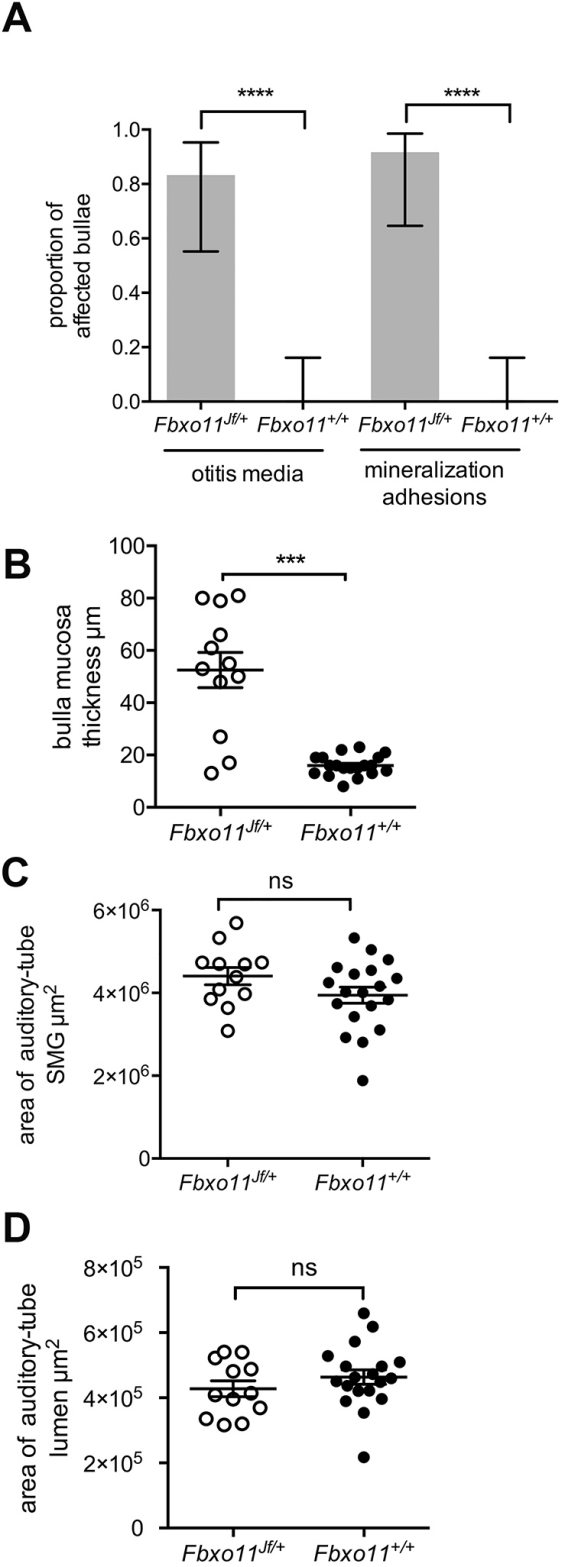
**Prevalence of otitis media in P105-P119 *Fbxo11* mice and morphometric analysis of auditory-tube SMGs and auditory tubes.** (A) The proportion of *Fbxo11^Jf/+^* bullae (*n*=12) with otitis media and mineralization and adhesions is high, but absent in *Fbxo11^+/+^* bullae (*n*=20). (B) *Fbxo11^Jf/+^* bulla mucosa is thickened. Cumulative areas of (C) auditory-tube SMGs and (D) auditory-tube lumen measured in 50-µm step sections are the same in *Fbxo11^Jf^^/+^* and *Fbxo11^+/+^* bullae. The histogram bar in A represents the mean and the error bars 95% confidence interval for each proportion. Frequency data were analyzed with Fisher's exact tests. Data in graphs B-D are represented as points; the error bars are mean±s.e.m. Data was analyzed with Student’s *t*-tests. Two-tailed tests: ns, not significant (*P*>0.05), ***P<0.001, ****P<0.0001.

We next investigated the onset of otitis media in *Fbxo11^Jf/+^* mice and key features of the mature lesion. The persistence of mesenchyme in *Fbxo11^Jf/+^* bullae at P21 coincides with the initiation of otitis media and the appearance of an inflammatory cell effusion of the bulla cavity (Fig. 4). By P57, remnant mesenchyme tissue is replaced by slender fibrous adhesions linking the mucosa with tympanic membrane, and foci of mucosal and tympanic membrane mineralization were present. These histological features were prevalent in P105-P119 *Fbxo11^Jf/+^* mice (Fig. 5) along with serous bulla effusions (Fig. S4) and a thickened bulla mucosa (Figs 4,5).

Adhesions between the mucosa and tympanic membrane cause indentations in the curved tympanic membrane (Fig. 4). There were a number of instances where adhesions and mineralized foci co-exist (Fig. 4N), suggesting that their formation is part of the same process. Cross-linked adhesions can form compartments containing effusions of varying cellularity and eosin staining intensity but, in 1 of 12 *Fbxo11^Jf/+^* bullae, there was no effusion (Fig. 4). Mineralized foci have the appearance of calcium deposits, but decalcification precludes histochemical confirmation (Fig. 4). There were ∼200 mineralized foci per bulla (a median of 0.74 per step section level) and ∼53% of section levels showed adhesions. Other histological findings in *Fbxo11^Jf/+^* mice included foci of persistent mesenchyme associated with tympanic membrane (2/12 bullae) (Fig. 4) and cholesterol granuloma (1/12 bullae). Some *Fbxo11^Jf/+^* mice with chronic otitis media had small populations of ciliated cells in the ordinarily non-ciliated promontory epithelium (Fig. 4). In addition, there was no extra-bulla soft-tissue mineralization in P61-P265 *Fbxo11^Jf/+^* (*n*=16) or P60-P265 *Fbxo11^+/+^* (*n*=6) and C57BL6/J (*n*=7) mice.

Hardisty et al. (2003) reported auditory-tube abnormalities, including auditory-tube epithelial cell sloughing and tube narrowing at the bulla ostia, in newborn *Fbxo11^Jf/+^* mice; in P50 *Fbxo11^Jf/+^* mice, a bend closer to the nasopharyngeal opening was seen. We observed similar features in wild-type mice. For instance, the normal auditory-tube epithelial lining can appear as a projecting sheet in a tangential plane of section and the auditory-tube tapers towards its entrance into the bulla (Fig. S1). Furthermore, formalin fixation can induce artefactual contraction of auditory-tube-associated muscle, producing a medial bend at its junction with the nasopharynx. We found no significant differences in the overall size of the auditory-tube lumen or in auditory-tube submucosal gland (SMG) size in *Fbxo11^Jf/+^* and *Fbxo11^+/+^* mice (Fig. 5).

### Otitis media in *Mecom*^*Jbo/+*^ mice is unrelated to a bulla cavitation defect

To further explore a link between bulla cavitation and the distinctive histopathology of otitis media, histopathology of *Fbxo11^Jf/+^* mice was compared to that of another established mouse model of chronic otitis media, *Mecom^Jbo/+^*. Otitis media was present in 10/14 P22 *Mecom^Jbo/+^* bullae, but there was no evidence of persistent mesenchyme (Fig. S3). A total of 10/14 P119-P133 *Mecom^Jbo/+^* bullae had suppurative otitis media, as previously described (Parkinson et al., 2006; Cheeseman et al., 2011), but there was no evidence of mucosal mineralization or adhesions. K8, K7/K19 and K5 are expressed in ciliated epithelium in *Mecom^Jbo/+^* and *Fbxo11^Jf/+^* bullae, in the hyperplastic P22 *Mecom^Jbo/+^* promontory epithelium, and in P13 *Fbxo11^Jf/+^* retained MAE (Fig. S3). There was no bulla pathology in P119-P133 wild-type *Mecom^+/+^* littermates (*n*=10). *Mecom^Jbo/+^* bulla exudates were more cellular and had higher neutrophil leukocyte (NL) differentials, higher bacterial-culture-positive rates, and higher numbers of foreign-body particles than *Fbxo11^Jf/+^* effusions (Fig. S4).

### Temporal expression of FBXO11 and its cargoes BCL6 and SNAI1

To gain additional insights into a role for FBXO11 in bulla mesenchyme physiology, we investigated the expression of *Fbxo11* and its putative cargoes *Snai1* and *Bcl6* in a time-course study. At E15.5, the first pharyngeal pouch epithelium had *Fbxo11* and *Bcl6* ISH signals, whereas *Snai1* was widely expressed in sub-epithelial connective tissue and otic capsule (Fig. S5). A strong *Bcl6* signal was present in dorsal epithelium of the nasopharynx (Fig. S5). From E17.5 to P9, *Fbxo11^Jf/+^* and *Fbxo11^+/+^* had comparable ISH signals for *Fbxo11*, *Snai1* and *Bcl6* in bulla mesenchyme, MAE and bulla mucosa, often with co-expression in individual cells (Fig. S5). *Bcl6* signals were higher in pseudostratified mucosal epithelium and MAE, whereas *Snai1* signals were greater in mucosal connective tissue. At P15, *Fbxo11*, *Snai1* and *Bcl6* signals were comparable in *Fbxo11^Jf/+^* and *Fbxo11^+/+^* bulla mucosa (Fig. S5). The persistent MAE in P15 and P21 *Fbxo11^Jf/+^* bullae had elevated signals for *Fbxo11*, *Snai1* and *Bcl6* (Fig. S5), but signals declined in P57 adhesion tissue and mucosa (Fig. S5).

## DISCUSSION

*FBXO11* is a candidate otitis media gene in humans (see discussion in Bhutta et al., 2017a,b), and we report that the established otitis media mouse model *Fbxo11^Jf/+^* (Hardisty-Hughes et al., 2006;Hardisty et al., 2003) has a bulla cavitation defect.

Lineage-tracing experiments in the mouse indicate that the non-ciliated epithelium of the attic and cochlea promontory arises from neural crest mesenchyme by MET. MET is reported to occur a few days after bulla cavitation with neural crest marker expression in attic epithelium evident at P16, and the expression of epithelial markers K14 at P16, laminin at P19 and E-cadherin at P20 (Thompson and Tucker, 2013). Our finding of a non-ciliated MAE in ≥E17.5 bullae is consistent with the Hilding et al. (1980) ultrastructural study in the rat. Bpifa1 is a highly expressed secreted protein that has previously been shown by IHC to stain the margin of the bulla mesenchyme, bulla epithelium and the medial surface of the tympanic membrane at P0, P5 and P10 (Mulay et al., 2018). The new ISH data indicates that Bpifa1 is a product of the MAE and bulla epithelium. In addition, we found that P1 MAE shows IHC staining with K8 and K7/K19 as well as K5. These keratins are expressed in primary air-liquid interface cultures of mouse middle ear epithelial cells that differentiate into ciliated and non-ciliated cells (Mulay et al., 2016). K5 is expressed in basal cells that give rise to ciliated and non-ciliated cells (Luo et al., 2017), although Tucker et al. (2018) found very few K5-positive cells in neural-crest-derived epithelium. Unlike the bulla pseudostratified epithelium, the MAE lacks a *Foxj1*-positive ciliated cell population and, taken together, it appears more likely that the MAE has a neural-crest origin.

Bpifa1 has an independent role in otitis media; its deficiency predisposes *Bpifa1* KO mice to late-onset otitis media (Bartlett et al., 2015), and Bpifa1 deficiency on a *Mecom^Jbo/+^* background increases the severity of otitis media (Mulay et al., 2018), but in neither case is there any indication that deficiency of Bpifa1 predisposes to otitis media via a bulla cavitation defect.

The mesenchyme shrinks during bulla cavitation and, as the MAE shortens, its cell density increases and its basal lamina remains in place. Ki67 labelling indicates a high MAE cell turnover and suggests the possibility that the squamous epithelium transitions into a compact, more cuboidal promontory epithelium. Thus, the non-ciliated epithelium in the bulla attic and promontory may conceivably arise through remodelling of a neural-crest-derived MAE formed by an embryonic MET event. The alternative scenario would entail postnatal MET seamlessly replacing the MAE and/or promontory epithelium. We found uniform K5 expression in P1 MAE and a population of K5-positive cells in newly formed promontory epithelium in P13 *Fbxo11^+/+^* mice, indicating a possible role for K5-positive putative stem cells in the normal development of non-ciliated epithelium.

There is an abrupt regression of bulla mesenchyme after P10 in the mouse and the bulla continues to enlarge until P21 (Park and Lim, 1992a). We found partial regression of mesenchyme in <10% of P11-P12 C57BL/6J bullae, which suggests that the process goes to completion within hours. In contrast to mice, mesenchyme occupies ∼20% of the bulla cavity at birth in normal human temporal bones and then disappears almost entirely by 1 year of age (Takahara et al., 1986); but, in children with congenital abnormalities (including Trisomy 13, Trisomy 18, Trisomy 21 and congenital heart defects), mesenchyme occupies ∼30% of the bulla cavity at birth and disappears by 3 years of age (Takahara and Sando, 1987).

Murine mesenchymal cells express AQP1 and αSMA at the time of regression, and these proteins may play a role in condensation of cells and ECM collagen fibrils. Aquaporin ion channels have roles in bulla mucosa physiology (Morris et al., 2012), and AQP1 in particular has a role in mesothelioma cell adhesion, interaction with ECM and cell migration (Jagirdar et al., 2016). The expression of αSMA in fibroblasts is associated with transdifferentiation into myofibroblasts (Stumm et al., 2014). There was no evidence of apoptosis and macrophage reaction being responsible for mesenchyme regression. The relatively constant number of mesenchyme cells present during regression suggests the possibility that these cells may be incorporated into the connective tissue of the newly formed promontory mucosa.

Defects in bulla cavitation are a cause of conductive hearing loss in *Tcof^+/−^* mice (Richter et al., 2010). In the *Cdh11* KO mouse, otitis media is associated with retained mesenchymal cells, and this may occur by altering the proportion of ciliated and non-ciliated epithelium and thereby predisposing to infection (Kiyama et al., 2018). Retention of mesenchyme in *Fbxo11^Jf/+^* mice is characterized by limited mesenchymal cell condensation and a failure of the MAE to shorten. The expression of αSMA and AQP1 by *Fbxo11^Jf/+^* mesenchymal cells persists after the normal window for regression and it is possible that mesenchymal cell motility is hindered by the onset of ECM fibrosis. Thereafter, mesenchymal cell numbers decline without detectable apoptosis. At P20, the MAE retains barrier function, forming a fluid-filled sac, and adhesions are likely to be initiated at points of contact with mucosa and tympanic membrane.

Foci of mucosal mineralization were described in 11-month-old *Fbxo11^Jf/+^* bullae (Hardisty et al., 2003) and, in the current study, we found evidence of early mucosal mineralization associated with fibrous adhesions. This unusual otitis media pathology has not been reported in other chronic otitis media mouse models (Bhutta et al., 2017b), nor did we observe it in serially sectioned *Mecom^Jbo/+^* bullae.

Foci of mucosal mineralization without adhesions are seen in *Rpl38^Ts/+^* mice, which have hyperphosphatemia (Noben-Trauth and Latoche, 2011), and focal calcification of the stapedial artery and otic capsule are reported in *Enpp1^asj/asj^* mice, which have reduced inorganic pyrophosphate (Tian et al., 2016). In both mutants, bulla mineralization is likely to have a metabolic basis and a metastatic presentation. Adhesions without mineralization are induced by intra-bulla bacterial challenge in the rat (Cayé-Thomasen et al., 1996; Cayé-Thomasen and Tos, 2002), guinea pig (Guan and Gan, 2013; Guan et al., 2013) and chinchilla (Guan et al., 2014). As focal mineralization appears to be restricted to the bulla mucosa, tympanic membrane and associated fibrous adhesions, it is likely to be dystrophic rather than a metastatic process in *Fbxo11^Jf/+^* mice and may be the result of necrosis of the remnant mesenchyme cells.

Serous bulla effusions were observed from P21 onwards in *Fbxo11^Jf/+^* mice but, at >P102, these become more suppurative. In addition, the ordinarily non-ciliated promontory epithelium develops a small population of ciliated cells that may have migrated from endoderm-derived epithelium or develop directly from hyperplastic neural-crest-derived epithelium (Tucker et al., 2018). Tucker et al. (2018) found very few K5-positive cells in healthy neural-crest-derived epithelium but larger numbers in inflamed promontory epithelium of *Tbx1*^+/−^ mice with otitis media. In keeping with this study, we found a large population of K5-positive cells in the hyperplastic promontory epithelium of P22 *Mecom^Jbo/+^* mice with otitis media. We observed similar patterns of K5, K7/K19 and K8 expression in ciliated epithelium in *Mecom^Jbo/+^* and *Fbxo11* bullae.

The cartilage that supports the dorsal region of the mouse auditory-tube develops at P1 (Park and Lim, 1992b); the tube lumen is normally closed, and its opening and closing is controlled by muscle action. As a result, characterization of the *in vivo* contours of the auditory-tube lumen is somewhat limited with formalin-fixed tissue sections. Furthermore, the 3D reconstruction of single tubes (Hardisty et al., 2003) may be prone to sample bias. We analyzed multiple tubes and found no change in the overall volume of tube lumen or enlargement of adjacent auditory-tube SMGs that would potentially compress the tube (Cayé-Thomasen and Tos, 2004). We did not recognize overt auditory-tube abnormalities in *Fbxo11^Jf/+^* mice and infer that bulla adhesions may contribute to the retention of effusion within the bulla cavity of *Fbxo11^Jf/+^* mice. An assessment of tube function in *Fbxo11^Jf/+^* mice is needed to assess the potential role of auditory-tube changes. One such approach is a dye clearance study that has elucidated the role of hypoplasia of veli palatini auditory-tube muscle in *Df1*/+ and *Tbx1^+/−^* mice with otitis media (Fuchs et al., 2015).

Duplex ISH showed that *Fbxo11* and its cargoes *Snai1* and *Bcl6* are colocalized in bulla mesenchyme, the MAE and in bulla mucosa from E17.5 to P11, and subsequently in both normal and defective mesenchyme regression. This indicates that there may be roles for FBXO11 in normal bulla development and homeostasis as well as in otitis media in *Fbxo11^Jf/+^* mice. The histology provides no clues that the bulla cavitation defect in *Fbxo11^Jf/+^* mice involves a role in modulation of Snai1 and MET/EMT as described in other systems (Díaz and de Herreros, 2016; Jin et al., 2015). The histological similarity of MAE in *Fbxo11^Jf/+^* and *Fbxo11^+/+^* mice suggests that (embryonic) MET proceeds normally in *Fbxo11^Jf/+^* mice. Furthermore, there was no evidence that the MAE or promontory epithelium lose epithelial cell marker (Bpifa1) expression or basal lamina, which would indicate EMT. The possibility of a postnatal MET seamlessly replacing the MAE and/or promontory epithelium is arguably less likely with a basal lamina in place. *Bcl6* is expressed in murine respiratory epithelium during embryonic (E17) development in the upper airway (Bajalica-Lagercrantz et al., 1998) and attenuates allergic airway inflammation (Seto et al., 2011), suggesting that perturbation of *Bcl6* may impact on inflammatory as well as developmental processes in *Fbxo11^Jf/+^* mice.

*FBXO11* and another TGFβ signalling pathway member, *TGIF*, are candidate disease genes in association studies of human patients with chronic otitis media with effusion (Bhutta et al., 2017a,b; Tateossian et al., 2013, 2015). We observed widespread nuclear localization of pSmad1/5 and pSmad2, indicative of TGF receptor signalling through BMPs and TGFβ, respectively, throughout normal mesenchyme regression in *Fbxo11^+/+^* and failed regression in *Fbxo11^Jf/+^* bullae. Although ISH data does not indicate obvious functional differences in TGF receptor signalling between genotypes, signalling may have important roles in the process. For example, in a mouse asthma model, TGFβ signalling induces transdifferentiation of lung fibroblasts to myofibroblasts and subsequently the balance of TGFβ/BMP modulates fibrosis (Stumm et al., 2014).

In conclusion, the MAE is present throughout the late embryonic period and mesenchyme regression in wild-type mice, and we infer that the MAE is remodelled into promontory epithelium. This interpretation is compatible with a neural crest origin of attic non-ciliated epithelium (Thompson and Tucker, 2013) but poses new questions about the timing of MET and the role of K5-positive putative stem cells in the MAE and its remodelling into promontory epithelium. Further lineage-tracing experiments are needed to clarify these questions. The mesenchyme and MAE appear similar in *Fbxo11^Jf/+^* and wild-type bullae until ∼P10, but then *Fbxo11^Jf/+^* mesenchyme fails to regress and this defective bulla cavitation is the initiating event of otitis media pathogenesis.

The normal developmental programme of the bulla may be orchestrated by the combined action of Snai1, Bcl6 and Smad transcriptional networks, and perhaps signalling between MAE and mesenchymal cells. The molecular mechanisms involved in bulla mesenchyme regression remain to be determined. Histological time-course studies have helped us to define the narrow window of mesenchyme regression, but have required large numbers of mice. *Ex vivo* culture of bullae and the use of signalling pathway inhibitors may be a useful alternative in future studies. Delayed bulla mesenchyme regression is easily recognized in the histology of weaning-aged mice and this criterion may reveal bulla cavitation defects in other established chronic otitis media mouse models. This work has potential clinical relevance in patients with FBXO11-associated chronic otitis media (Segade et al., 2006; Rye et al., 2011a,b, 2012; Bhutta et al., 2017a,b). In particular, it identifies new diagnostic criteria (adhesions associated with a non-metabolic cause of bulla soft-tissue mineralization) to suspect that a bulla cavity defect is an underlying cause of otitis media. In addition, techniques such as measuring tympanic membrane motion using scanning laser Doppler vibration (Wang et al., 2016) may be useful to identify immobilizing adhesions.

## MATERIALS AND METHODS

### Mice and husbandry

The animal experiments were reviewed and agreed by the Roslin Institute Animal Welfare and Ethical Review Body, and were performed under the authority of an appropriate UK Home Office Licence.

Heterozygous *Fbxo11^Jf/+^* mice (MGI, 1862017; European Mouse Mutant Archive, EM:00375) and their *Fbxo11^+/+^* wild-type littermates were generated by inter-crossing F1 *Fbxo11^Jf/+^* C57BL/6J C3H/HeH males with C57BL/6J (Charles River) females. Embryos were generated by timed matings with the day of plug designated as E0.5, or by IVF using *Fbxo11^Jf/+^* sperm and C57BL/6J oocytes and transfer into C57BL/6J recipient females. Newborn litters were designated P0 on the day of birth, and therefore age is accurate to ±12 h. Heterozygous *Mecom^Jbo/+^* mice and their wild-type littermate controls, *Mecom^+/+^*, are congenic on a C3H/HeH genetic background (MGI, 2158381; EMMA EM:00091). C57BL/6J (Charles River) mice also were bred for experiments. We analyzed whole litters of mice containing males and females in all the experiments.

Mice were housed in individually ventilated cages and their husbandry, microbial surveillance and health status are reported elsewhere (Azar et al., 2016). Genotyping was performed by Transnetyx using real-time PCR with the SNP assays for Fbxo11-1 Mut (*Fbxo11^Jf/+^* A1472T transversion; Hardisty-Hughes et al., 2006) and Mecom-1 Mut (*Mecom^Jbo/+^* A2318T transversion; Parkinson et al., 2006).

### Histology, immunohistochemistry and *in situ* hybridization

Pregnant females were euthanized by cervical dislocation and embryos were decapitated. Adult mice (≥P9) were euthanized using a rising concentration of CO_2_ or, alternatively, younger mice were euthanized by intraperitoneal (i.p.) injection of barbiturate. After decapitation, the head was skinned and the cranial vault was removed with a scalpel and the brain removed. Heads were immersion fixed in neutral buffered formalin and decalcified with 14% EDTA at room temperature on a roller mixer. Bulla histology was assessed in 4-µm wax sections stained with haematoxylin and eosin (H&E) or Picrosirius Red. Dorsal plane serial sections were made of the skull base of P105-P119 *Fbxo11^Jf/+^* (*n*=6) and *Fbxo11^+/+^* (*n*=10) littermates, and P22 *Mecom^Jbo/+^* (*n*=7), P119-P133 *Mecom^Jbo/+^* (*n*=7) and wild-type *Mecom^+/+^* littermates (*n*=10). P22 bullae were sectioned in 20-µm steps and older mice in 50-µm steps. The bullae were scored for the presence (or absence) of otitis media (based on the diagnostic criteria of fluid effusion in the bulla cavity and mucosal thickening with inflammatory cell infiltration); presence of adhesions and mineralized foci; and the number of foreign-body particles (plant-based material or hair shaft). In *Fbxo11^Jf/+^* bullae, individual adhesions occur at multiple section levels, whereas mineralized foci were often, but not always, small and discrete. Consequently, an additional count was made of mineralized foci in each step section.

*Fbxo11^Jf/+^* females (*n*=3 P61 and *n*=6 P119 mice), *Fbxo11^Jf/+^* males (*n*=3 P223 and *n*=4 P265 mice), *Fbxo11^+/+^* females (*n*=3 P60, *n*=3 P119 mice) and P265 C57BL/6J females (*n*=7) were examined for evidence of soft-tissue mineralization in predisposed sites such as whisker pad vibrissa follicle sheath, dorsal thorax skin, tongue, kidney, heart and testis (artery) in single H&E-stained section for each tissue.

*Fbxo11* and C57BL/6J mice from 1-4 pregnancies for each time point were collected for IHC and ISH studies (E15.5, *n*=3 *Fbxo11^Jf/+^*, *n*=4 *Fbxo11^+/+^*; E17.5, 1 *Jf/*+, 6 +/+; E18.5, 3 *Jf/*+, 7+/+; P1, 5 *Jf/*+, 7 +/+; P3, 2 *Jf/*+, 12 +/+; P5, 2 *Jf/*+, 4 +/+; P7, 2 *Jf/*+, 6 +/+; P9, 3 *Jf/*+, 8 +/+; P10, 10 *Jf/*+, 16 +/+; P11, 8 *Jf/*+, 8 +/+; P12, 2 *Jf/*+, 14 +/+; P13, 6 *Jf/*+, 6 +/+; P15, 4 *Jf/*+, 5 +/+; P21, 5 *Jf/*+, 2 +/+; P57, 3 *Jf/*+, 6 +/+; P10 C57BL/6J *n*=22; P11 C57BL/6J *n*=33; P12 C57BL/6J *n*=14). Embryo heads were fixed whole, or the brain was removed in postnatal mice. Heads were formalin fixed for 18-24 h and embryo and P1 mouse skulls were processed for wax sections without decalcification. P3-P7 heads were decalcified for 8 h, P9-P13 heads for 24 h, P15-P21 heads for 48 h and P57 heads for 72 h. Decalcified skull base samples were trimmed, wax embedded, then sectioned to expose the bulla cavity. Tissue arrays were made by re-blocking groups of skulls and enabled whole litters to be assessed in a single slide. The skull ID can be made unambiguous from its position in the array and specimen orientation (rostral facing left or right); the example in Fig. S6 shows P10 C57BL/6J skulls (*n*=7).

IHC and ISH were performed on a Leica Bond Rx machine; antigen retrieval for IHC was performed using a Histos microwave machine unless otherwise stated. ISH was performed using Advanced Cell Diagnostics LS 2.5 RNAScope probes and Leica detection kits according to the manufacturer's instructions (see Table S1 for reagents and protocols). We note that the *Fbxo11* probe has 20 primers that target base pairs 662-1675. Although the *Fbxo11^Jf/+^* mutation, an AT transversion in exon 13 at position 1472, occurs within this region (Hardisty-Hughes et al., 2006), the *Fbxo11* hybridization signal in *Fbxo11^Jf/+^* and *Fbxo11^+/+^* mice appears comparable.

Bright-field images were acquired on an Olympus BX41 microscope equipped with a DP72 camera and Cell D software. Slide scans were made using a Hamamatsu NanoZoomer and analyzed with NanoZoomer software and Qu-Path software (Bankhead et al., 2017). Bulla mucosa thickness, cumulative area of auditory-tube SMGs and auditory-tube lumen profiles were measured in 50 µm step sections of P105-P119 *Fbxo11^Jf/+^* and *Fbxo11^+/+^* mice. Representative skull sections were used to measure bulla mesenchyme area rostral to the malleus/stapedial artery, and mesenchymal cell and MAE cell (nuclei) densities. The length of the MAE was expressed as a ratio to the margin of the underlying cochlea promontory bone, which closely approximates to promontory epithelium length.

### *In vivo* labelling with FITC–dextran-70

P20 *Fbxo11^Jf/+^* mice were labelled with FITC–dextran-70 (Sigma Aldrich) by i.p. injection of 100 µl of 25 mg/ml of reagent dissolved in sterile PBS. Mice were euthanized 60 min after injection and a post-mortem tail tip collected for genotyping. In order to minimize tissue contamination with blood-borne FITC–dextran-70, initial dissection was limited to the removal of the cranial vault to aid fixative penetration. After fixation for 48-72 h, the heads were decalcified for 72 h, trimmed and wax sections used for anti-FITC IHC (Table S1).

### Microbiology and cytology of the nose and bullae

Post-mortem bulla and nasal washes were collected from P105-P119 *Fbxo11^Jf/+^* (*n*=11), *Fbxo11^+/+^* littermate/cagemate controls (*n*=10), P98-P147 *Mecom^Jbo/+^* (*n*=8) and *Mecom^+/+^* littermate/cagemate controls (*n*=9) for Giemsa-stained cytospin cytology preparations and for quantitative aerobic bacterial culture as previously described (Azar et al., 2016).

### Transmission electron microscopy

For transmission electron microscopy (TEM), the dissected skull base of P0 C57BL/6J mice was fixed in 3% glutaraldehyde in 0.1 M sodium cacodylate buffer, pH 7.3, for 2 h then washed in three 10 min changes of 0.1 M sodium cacodylate. Specimens were then post-fixed in 1% osmium tetroxide in 0.1 M sodium cacodylate for 45 min, then washed in three 10 min changes of 0.1 M sodium cacodylate buffer. These samples were then dehydrated in 50%, 70%, 90% and 100% ethanol (×3) for 15 min each, then in two 10 min changes in propylene oxide. Samples were then embedded in TAAB 812 resin. Sections of 1 μm thickness were cut on a Leica Ultracut ultramicrotome, stained with Toluidine Blue, and viewed on a light microscope to select suitable areas for investigation. Ultrathin sections, 60 nm thick, were cut from selected areas, stained in uranyl acetate and lead citrate then viewed in a JEOL JEM-1400 Plus TEM. Representative images were collected on a GATAN OneView camera.

### Statistical analysis

The statistical tests were chosen after performing D'Agostino and Pearson omnibus normality tests. The summary statistics and the statistical tests used for each data set are described in the figure legends. Two-tailed tests were used throughout and test values of *P*<0.05 were considered to be statistically significant. Graphs and statistics were generated using GraphPad Prism.

## Supplementary Material

Supplementary information
