# Supplementary material for: Chronic otitis media is initiated by a bulla cavitation defect in the FBXO11 mouse model
Source: Dis Model Mech. 2019 Mar 21;12(3):dmm038315. doi: 10.1242/dmm.038315 (PMC6451434; doi:10.1242/dmm.038315)
Supplement: Supplementary information [file dmm-12-038315-s1.pdf]

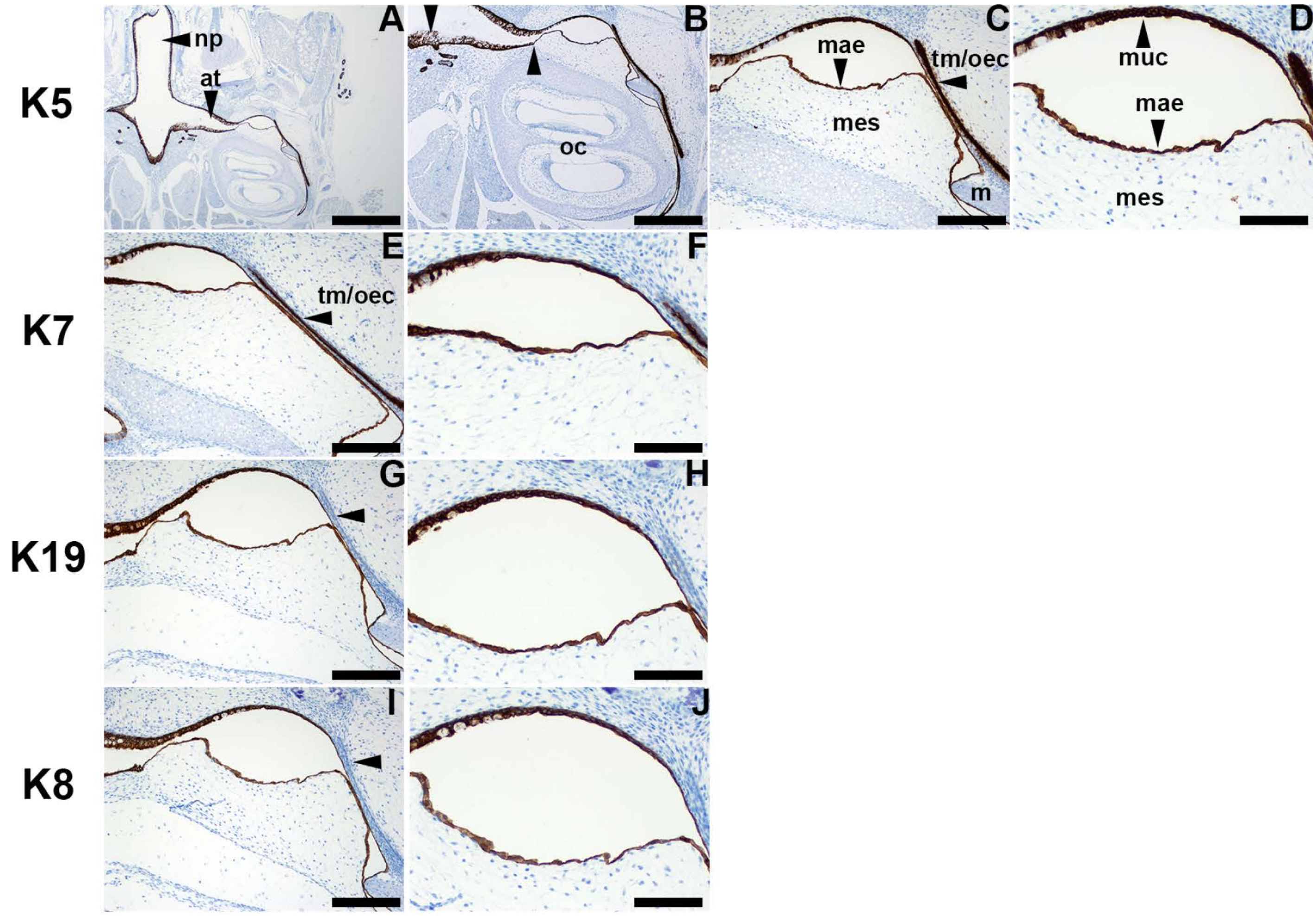


**Fig. S1. Expression of keratins 5,7,19,8 in MAE**

P1 *Fbxo11*+/+ (mixed C57BL/6J C3H background) mouse. (A-D) Epithelium of the nasopharynx, auditory-tube, bulla mucosa and MAE stain positively for K5 by IHC. Panels A,B,C and D are successively higher magnification images of the same section. The MAE and the pseudostratified ciliated epithelium of the bulla mucosa stain positively for K7, K19 and K8. Note the future tympanic membrane and outer ear canal epithelia stain with K5 (C) and K7 (E), but not with K19 (G arrowhead) or K8 (I arrowhead). Note panel pairs (C,D), (E,F), (G,H) and (I,J) are low and high magnification images of the same section.

Note panel B auditory-tube epithelial cells in tangential plane of section (downward arrowhead) and the tube tapers towards the entrance into the bulla (upward arrowhead).

Abbreviations: (at) auditory-tube, (oc) otic capsule, (m) malleus, (mae) mesenchyme-associated epithelium, (mes) mesenchyme, (muc) mucosa, (np) nasopharynx, (tm/oec) tympanic membrane/outer ear canal epithelium.

Scale bars: (A) = 1 mm; (B) = 500 μm; (C,E,G,I) = 200 μm; (D,F,H,J) = 100 μm.

Disease Models & Mechanisms • Supplementary information

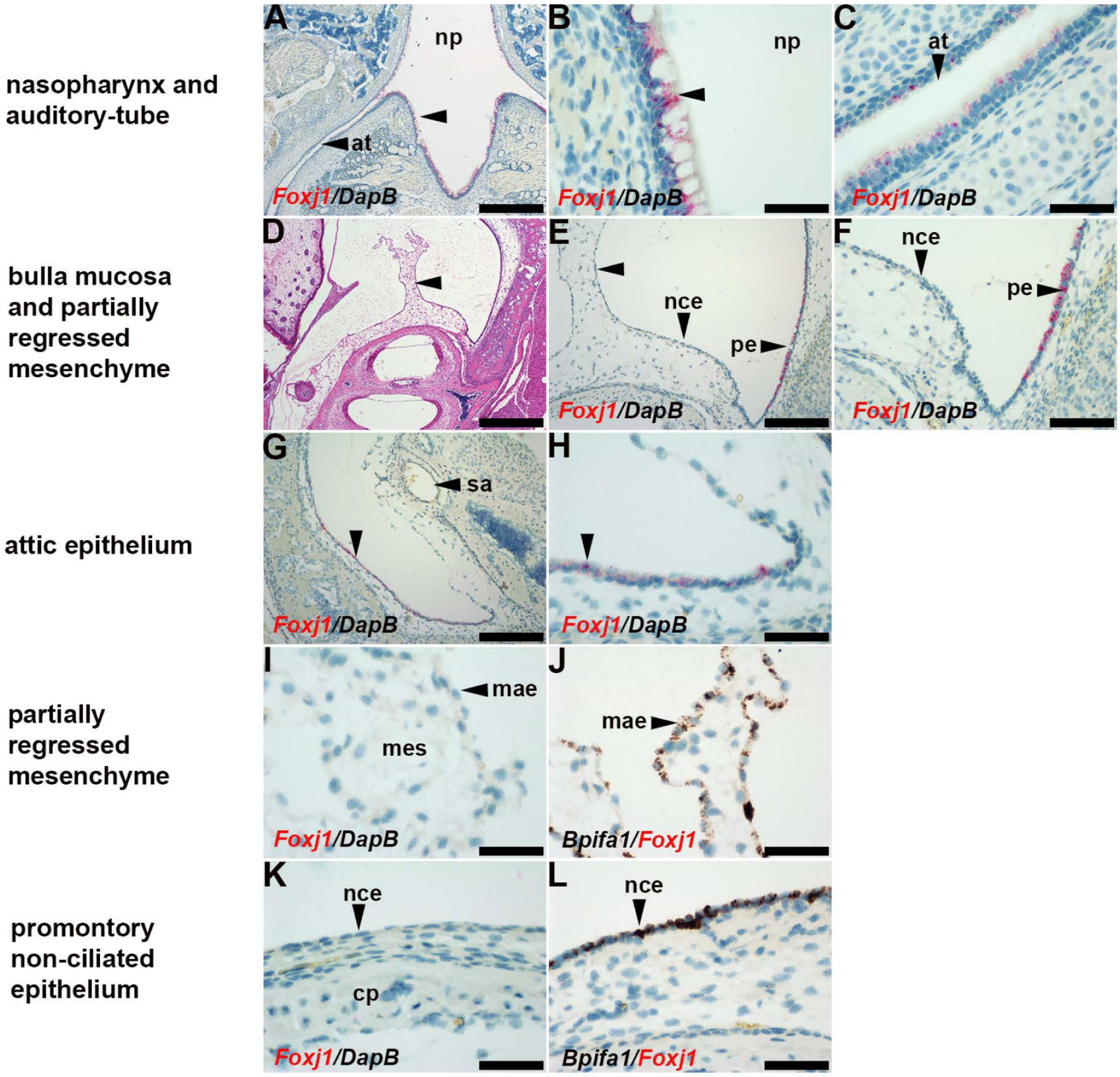


**Fig. S2. *Foxj1*-positive ciliated cells are absent in the MAE and non-ciliated cochlea promontory epithelium**
P11 C57BL/6J (A-C,E-I,K) Duplex ISH with *Foxj1* channel 2 probe signals are red spots and *DapB* negative control channel 1 probe (note that brown spots are absent). (A-C) Nasopharynx and auditory-tube have ciliated cells with *Foxj1* signals (unlabelled arrowheads); higher magnification (B) nasopharynx with ciliated and goblet cell populations and (C) auditory-tube. (D) H&E stained section of bulla with partial regression of mesenchyme over cochlea promontory (unlabelled arrowheads in D and E); higher magnification panels E and F showing *Foxj1* signals in pseudostratified mucosal epithelium but are absent in non-ciliated promontory epithelium. (G,H) Attic epithelium has *Foxj1*-positive ciliated cells (unlabelled arrowheads), (H) is higher magnification image of G. (I,J) Partially regressed bulla mesenchyme.
(I) Duplex ISH for *Foxj1/DapB* shows *Foxj1* signals are absent from the MAE, (J) duplex *Bpifa1/Foxj1* ISH shows *Bpifa1*-postive MAE. (K,L) Using the same ISH probe pairs, newly formed cochlea promontory epithelium are (K) *Foxj1*-negative and (L) *Bpifa1*-positive.
Abbreviations: (at) auditory-tube, (cp) cochlea promontory, (mae) mesenchyme-associated epithelium, (mes) mesenchyme, (muc) mucosa, (nce) non-ciliated epithelium, (np) nasopharynx, (pe) pseudostratified epithelium, (sa) stapedial artery.
Scale bars: (A,D) = 500 µm; (E,G) = 200 µm; (F,H) = 100 µm (B,C,I-L) = 50 µm.

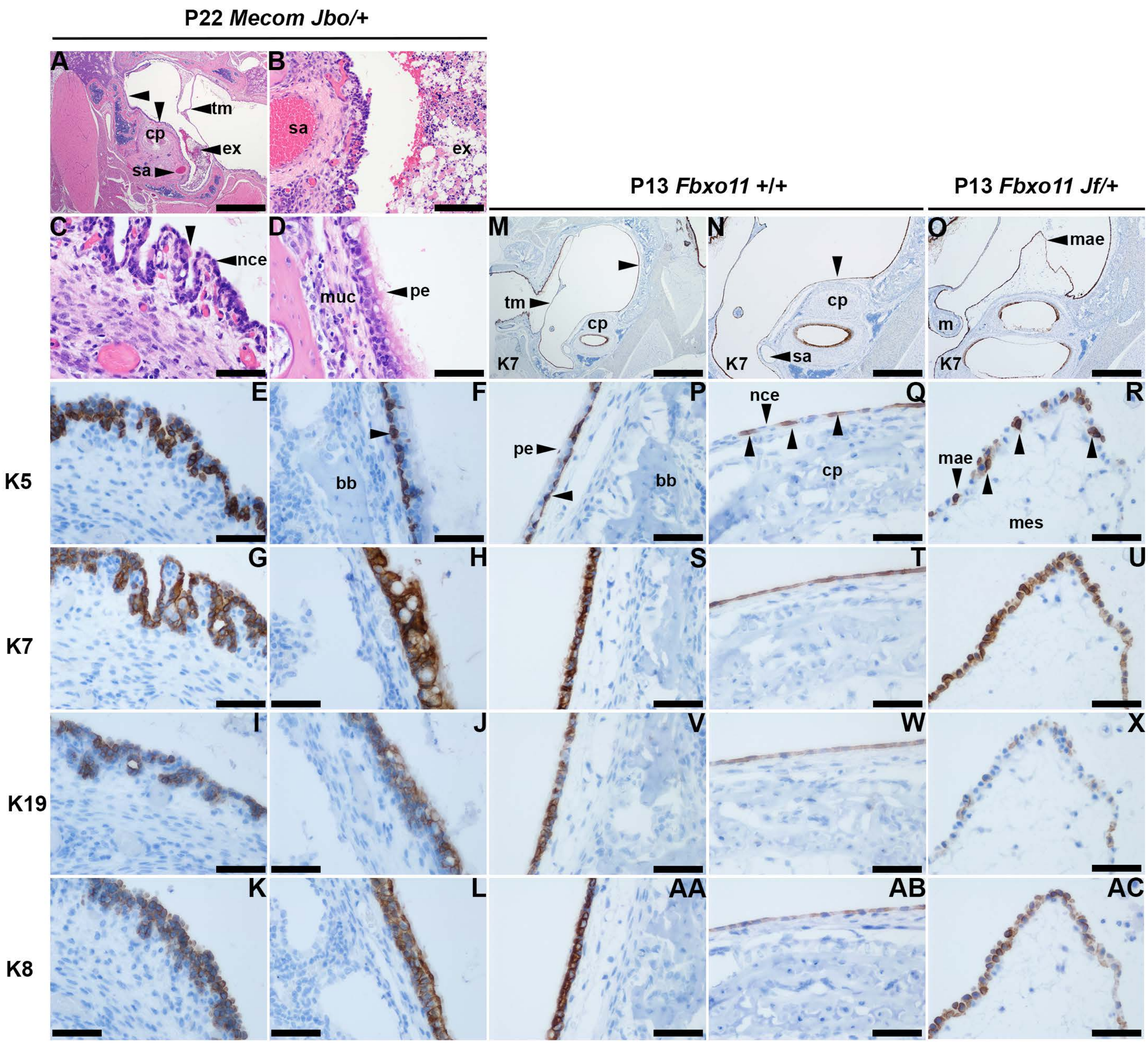


**Fig. S3. Otitis media in *Mecom Jbo/+* and keratin expression in *Mecom Jbo/+* and *Fbxo11* bullae**

(A-L) P22 *Mecom Jbo/+* bulla shows no evidence of remnant mesenchyme and the cavity contains inflammatory cell exudate; vertical and horizontal unlabelled arrowheads indicate site of the non-ciliated promontory epithelium and ciliated mucosa respectively. (B) Higher magnification of the hyperplastic promontory mucosa adjacent to the stapedial artery; the lumen contains exudate with apoptotic cells. (C) The hyperplastic promontory epithelium is mostly non-ciliated but has the occasional ciliated cell (vertical arrowhead). (D) Pseudostratified ciliated epithelium. Keratin IHC staining of promontory epithelium (E,G,I,K) and pseudostratified epithelium (F,H,J,L). Note K5-positive cells in hyperplastic promontory epithelium and basal cell staining in ciliated epithelium (arrowhead). K7, K19 and K8 staining is widespread in each epithelium. (M-AC) P13 *Fbxo11* mice. (M,N,O) K7 IHC. (M,N) *Fbxo11 +/+* bulla that has undergone normal mesenchyme regression, panels are different magnifications of the same section; horizontal unlabelled arrowhead indicates site of the ciliated epithelium shown in panels (P,S,V,AA), vertical unlabelled arrowhead the site of the non-ciliated epithelium of the cochlea promontory in panels (Q,T,W,AB). (O) *Fbxo11 Jf/+* remnant mesenchyme and MAE shown at higher magnification in panels (R,U,X,AC). Note K5, K7, K19 and K8 IHC staining of pseudostratified ciliated epithelium is similar in *Fbxo11 Jf/+* and *Fbxo11 +/+* bullae and only the latter is shown. (P-R) K5-positive basal cells in ciliated epithelium (unlabelled arrowhead) and K5 staining in subsets of *Fbxo11 +/+* promontory epithelium (unlabelled arrowheads) and remnant *Fbxo11 Jf/+* MAE (unlabelled arrowheads). (S-U) K7; (V-X) K19; and (AA,AB,AC) K8 staining is more uniform for each epithelium respectively, K19 gives the the weakest signal.

Abbreviations: (bb) bulla bone (cp) cochlea promontory, (ex) inflammatory cell exudate, (m) malleus, (mae) mesenchyme-associated epithelium, (mes) mesenchyme, (muc) mucosa, (nce) non-ciliated epithelium, (sa) stapedial artery, (tm) tympanic membrane.

Scale bars: (A,M) = 1 mm; (N,O) = 500 µm (B) = 100 µm; (C-L, P-AC) = 50 µm.

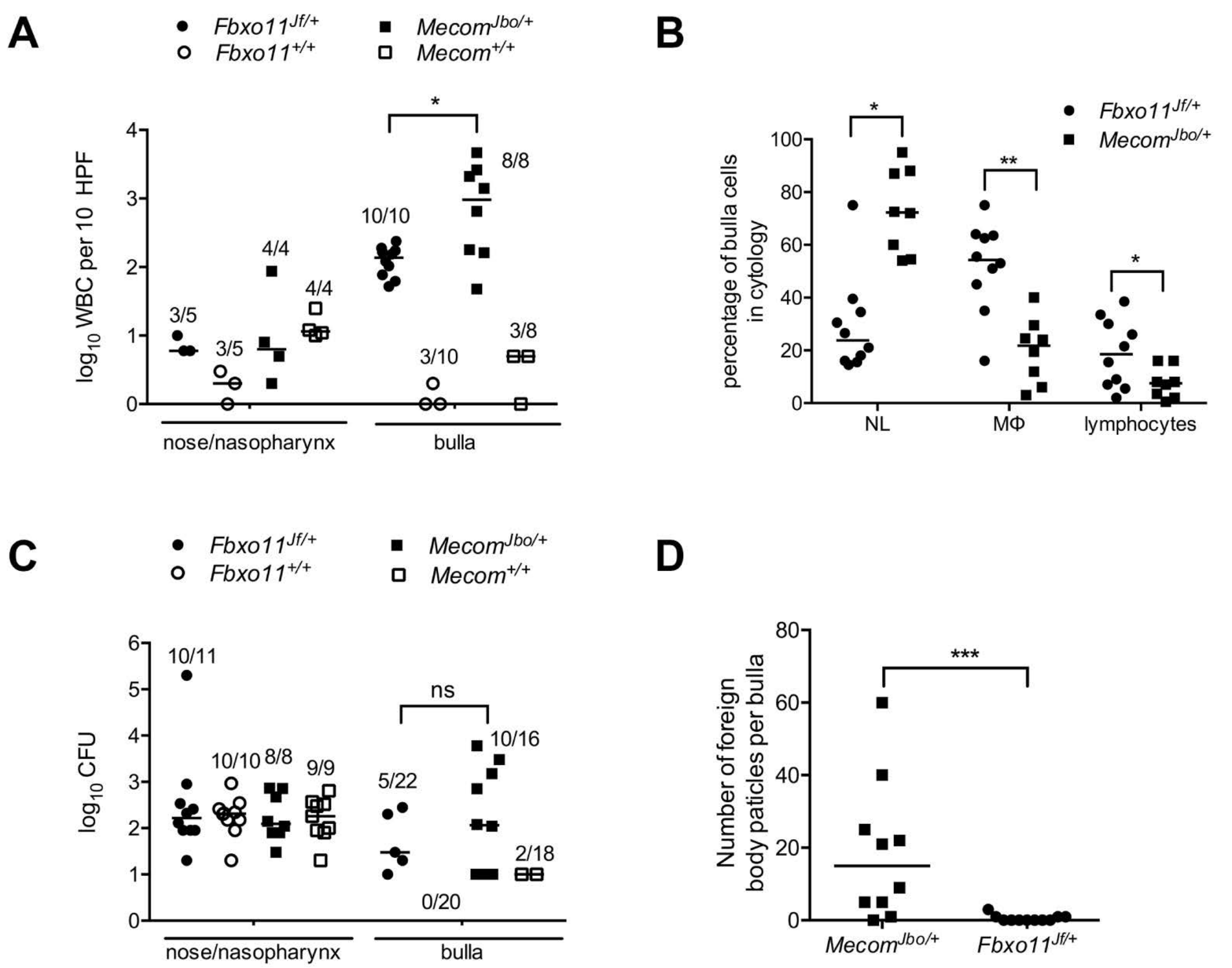


**Fig. S4. *Fbxo11 Jf/+* compared to *MecomJbo/+* mice have serous, non-suppurative otitis media**
(A) Nose/nasopharynx and bulla WBC counts, (B) bulla cytology differentials and (C) nose/nasopharynx and bulla bacterial CFU. (A) WBC counts are lower in P105-P119 *Fbxo11 Jf/+* than P98-P147 *Mecom Jbo/+* bulla fluids. (B) Cytology differentials show macrophages (MØ) and lymphocytes predominate in *Fbxo11 Jf/+* bulla effusions whereas neutrophils (NL) predominate in *Mecom Jbo/+* bulla exudates. (C) Bacterial CFU are comparable in the nasal passages and bullae. Data are represented as points and the median. The ratio beside each set of data in panels A and C represents the number of positive cases/the total number tested. Bacterial isolates from nasal washes and bullae included: *Enterococcus* sp., *Gemella* sp., DNAase negative *Staphylococcus* sp., *Pasteurella* sp., *Proteus mirabilis*, *Stenotrophomonas maltophila*, *E.coli* and a Gram-positive bacillus. (D) Foreign body particles in the bulla were lower in *Fbxo11 Jf/+* bullae ($n$=11) compared to *Mecom Jbo/+* bullae ($n$=10). Two-tailed Mann-Whitney tests were used to analyze bulla WBC, WBC differentials and CFU data (excluding zero values for cell counts and culture negative results) and foreign body particle data. ns, not significant ($P$>0.05), * $P$<0.05, ** $P$<0.01, ***$P$<0.001.

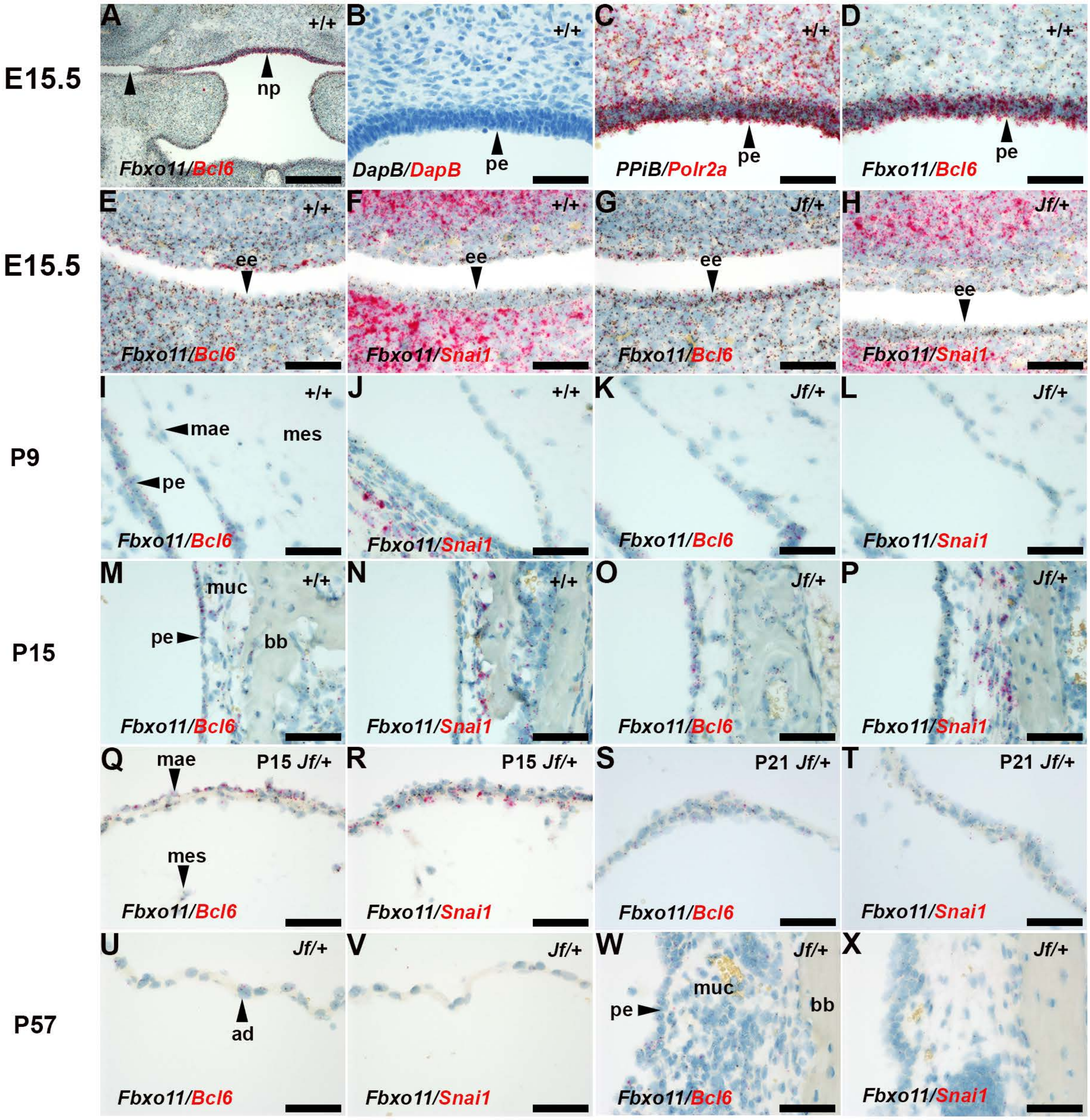


**Fig. S5. Embryonic and postnatal expression of *FBXO11* and its cargoes *SNAI1* and *BCL6***
Duplex ISH with *Fbxo11* channel 1 (C1) probe signals are brown spots, and *Bcl6* and *Snai1* channel 2 (C2) probes signals are red spots. The probe pairs and genotype are indicated in each panel. (A-H) E15.5 mice. (A-D) Nasopharynx, (E-H) first pharyngeal pouch (unlabelled arrowhead in A). (A,D) *Fbxo11/Bcl6* ISH, nasopharynx epithelium has an intense signal for *Bcl6*. (B) Duplex negative control probe *DapB*, note brown and red spots are absent, and (C) positive duplex control probes *PPiB*-C1 and *Polr2ra*-C2. (E,F; I,J; M,N) *Fbxo11 +/+* and (G,H; K,L; O,P) *Fbxo11 Jf/+* have comparable *Fbxo11*, *Bcl6* and *Snai1* signals for each tissue at E15.5, P9 and P15 respectively with examples of cellular co-localization of *Fbxo11/Bcl6* and *Fbxo11/Snai1*. *Bcl6* signals are greater in the epithelium whereas the *Snai1* signals are greatest in submucosal connective tissue. (I-L) P9 bulla mesenchyme, MAE and mucosa. (M-P). P15 bulla mucosa. (Q,R) Remnant MAE in P15 and (S,T) P21 *Fbxo11 Jf/+* bullae. (U-X) P57 *Fbxo11 Jf/+* (U,V) adhesion tissue and (W,X) mucosa.
Abbreviations: (ad) adhesion, (bb) bulla bone, (ee) endoderm derived epithelium, (mae) mesenchyme-associated epithelium, (mes) mesenchyme, (muc) mucosa, (pe) pseudostratified epithelium, (+/+) *Fbxo11 +/+*, (*Jf*/+) *Fbxo11 Jf/+*.
Scale bars: (A) = 200 µm; (B-X) = 50 µm.

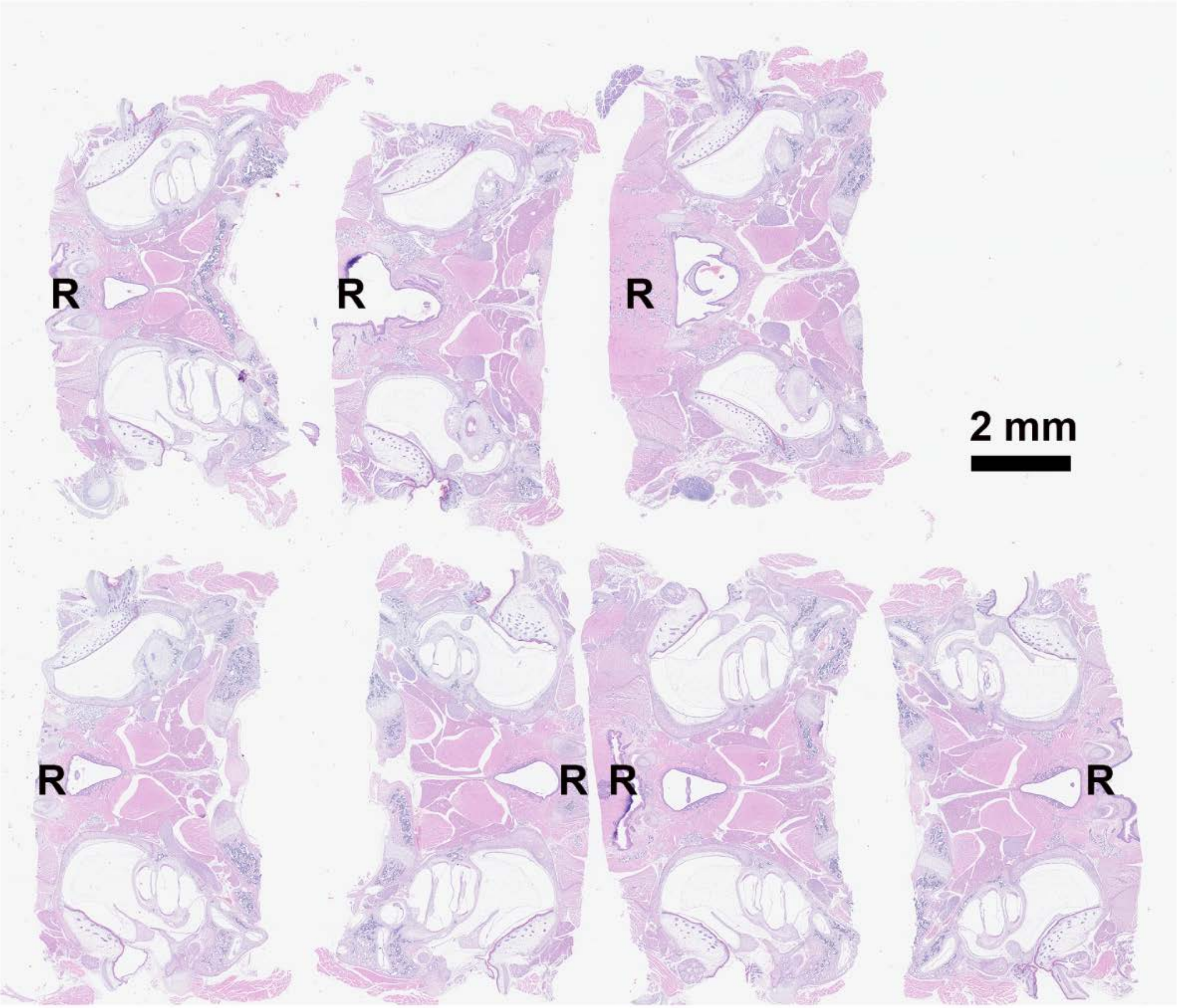


**Figure S6. Histological array of P10 C57BL/6J heads**
Multi blocked skulls, note the unambiguous ID of each sample is designated by the position in the array and specimen orientation, rostral (R) surface facing left or right.
Image from slide scan.

Disease Models & Mechanisms • Supplementary information

**TABLE S1. REAGENTS AND PROTOCOLS**

| Antibody | Details | Host species | Antigen retrieval | AR Temperature/time | Dilution | Incubation | Secondary reagent | Chromogen |
|---|---|---|---|---|---|---|---|---|
| | | | | | | | | |
| Aquaporin 1 | Abcam ab168387 | Rabbit | Citrate buffer pH 6.0 | Histos MW 110°C 5 mins | 1/300- 1/400 | o/n 4°C | Envision Rabbit 40 mins | Vector Nova Red |
| Cleaved caspase 3 | Abcam ab2302 | Rabbit | EDTA pH8.0 | Histos MW 60°overnight | 1/20 | 60 mins r/t | Envision Rabbit 40 mins | DAB |
| Cleaved caspase 3 (Asp175) (D3E9) | Cell Signaling #9579 | Rabbit | Citrate buffer pH 6.1 | Histos MW 110°C 5 mins | 1/251 | o/n 4°C | Envision Rabbit 40 mins | DAB |
| wide spectrum Cytokeratin | Abcam ab9377 | Rabbit | Proteinase K | Bond RX 37°C 15 mins | 1/150 | 30 mins r/t | Envision Rabbit 40 mins | DAB |
| Keratin 5 [EP1601Y] | Abcam 52635 | Rabbit | Tris-EDTA buffer pH 9.0 | Histos MW 110°C 5 mins | 1/200 | o/n 4°C | Envision Rabbit 40 mins | DAB |
| Keratin 7 [EP17078] | Abcam 181598 | Rabbit | Tris-EDTA buffer pH 9.1 | Histos MW 110°C 5 mins | 1/8000 | o/n 4°C | Envision Rabbit 40 mins | DAB |
| Keratin 8 [EP1628Y] | Abcam 53280 | Rabbit | Tris-EDTA buffer pH 9.2 | Histos MW 110°C 5 mins | 1/250 | 30 mins r/t | Envision Rabbit 40 mins | DAB |
| Keratin19 [EP1580Y] | Abcam 52625 | Rabbit | Tris-EDTA buffer pH 9.5 | Histos MW 110°C 5 mins | 1/600 | o/n 4°C | Envision Rabbit 40 mins | DAB |
| E Cadherin | Abcam ab76319 | Rabbit | Lieca bond ER1 | Bond RX 100°C 20 mins | 1/300 | 30 mins r/t | Envision Rabbit 40 mins | DAB |
| F4/80 | Bio RAD MCA497G | Rat | Proteinase K | Bond RX 37°C 15 mins | 1/600 | 30 mins r/t | Vector Immpress anti Rat 15 mins r/t | DAB |
| FITC | Abcam ab49368 | rabbit | None required | N/A | 1/100 | 30 mins r/t | Goat anti-rabbit (Sigma A3821) 1/50 30 mins r/t | ImmPACT Vector Red Alk P (SK-5105) substrate kit 2-10 minutes r/t |
| Ki 67 | Abcam ab15580 | Rabbit | Citrate buffer pH 6.0 | Histos MW 110°C 5 mins | 1/1000 | 30 mins r/t | Envision Rabbit 40 mins | DAB |
| Laminin | Abcam ab11575 | Rabbit | EDTA pH 8.0 | Histos MW 60°overnight | 1/300 | o/n 4°C | Envision Rabbit 40 mins | Vector Nova Red |
| Phospho SMAD1/SMAD5 Ser4653 Ser465 | Thermo Fisher 41H14L11 | Rabbit | Citrate buffer pH 6.2 | Histos MW 100°C 20 mins | 1/200 | o/n 4°C | Envision Rabbit 40 mins | DAB |
| Phospho SMAD2 Ser465 Ser467 | Thermo Fisher 44-244G | Rabbit | Citrate buffer pH 6.1 | Histos MW 100°C 20 mins | 1/600 | o/n 4°C | Envision Rabbit 40 mins | DAB |
| Smooth muscle actin | Abcam ab5694 | Rabbit | Citrate buffer pH 6.0 | Histos MW 110°C 5 mins | 1/150 | 30 mins r/t | Envision Rabbit 40 mins | DAB |
| Vimentin EPR3776 | Abcam ab92547 | Rabbit | Citrate buffer pH 6.0 | Histos MW 100°C 20 mins | 1/800 | 30 mins r/t | Envision Rabbit 40 mins | DAB |

| ISH | 2.5 LS probes | Notes |
|---|---|---|
| Mm-Fbxo11 | RNAScope 497008 | |
| Mm-Snai1-C2 | RNAScope 451218-C2 | |
| Mm-Bcl6-C2 | RNAScope 455318-C2 | |
| Mm-Bpifa1 | RNAScope 512598 | amp 9 reduced to 5 mins |
| Mm-Foxj1-C2 | RNAScope 317098-C2 | |
| Negative Control Probe DapB | RNAScope 312038 | |
| Duplex Negative Control Probe (DapB-C1, DapB-C2) | RNAScope 320758 | |
| Duplex Control Probes (Mm-PPIB-C1, Mm-Polr2a-C2) | RNAScope 320768 | |

| | |
|---|---|
| RNAscope® 2.5 LS Duplex Reagent Kit- (RED/BROWN) | 322440 |
